# Single-cell sequencing of full-length transcripts and T-cell receptors with automated high-throughput Smart-seq3

**DOI:** 10.1186/s12864-024-11036-0

**Published:** 2024-11-21

**Authors:** Hsiu-Chun Chuang, Ruidong Li, Huang Huang, Szu-Wen Liu, Christine Wan, Subhra Chaudhuri, Lili Yue, Terence Wong, Venina Dominical, Randy Yen, Olivia Ngo, Nam Bui, Hubert Stoppler, Tangsheng Yi, Silpa Suthram, Li Li, Kai-Hui Sun

**Affiliations:** Gilead Sciences, Inc., 333 Lakeside Drive, Foster City, CA 94403 USA

**Keywords:** High-throughput Smart-seq3, 10X, Single-cell RNA sequencing, Single-cell TCR sequencing

## Abstract

We developed an automated high-throughput Smart-seq3 (HT Smart-seq3) workflow that integrates best practices and an optimized protocol to enhance efficiency, scalability, and method reproducibility. This workflow consistently produces high-quality data with high cell capture efficiency and gene detection sensitivity. In a rigorous comparison with the 10X platform using human primary CD4 + T-cells, HT Smart-seq3 demonstrated higher cell capture efficiency, greater gene detection sensitivity, and lower dropout rates. Additionally, when sufficiently scaled, HT Smart-seq3 achieved a comparable resolution of cellular heterogeneity to 10X. Notably, through T-cell receptor (TCR) reconstruction, HT Smart-seq3 identified a greater number of productive alpha and beta chain pairs without the need for additional primer design to amplify full-length V(D)J segments, enabling more comprehensive TCR profiling across a broader range of species. Taken together, HT Smart-seq3 overcomes key technical challenges, offering distinct advantages that position it as a promising solution for the characterization of single-cell transcriptomes and immune repertoires, particularly well-suited for low-input, low-RNA content samples.

**Supplementary Information:**

The online version contains supplementary material available at 10.1186/s12864-024-11036-0.

## Background

Single-cell RNA-seq (scRNA-seq) provides valuable insights into cellular heterogeneity, and single-cell TCR sequencing (scTCR-seq) captures paired alpha (TRA) and beta (TRB) chain information, enabling precise identification of T-cell clonotypes and their corresponding antigen specificities [1, 2]. Advancements in single-cell technologies allow for concurrent profiling of the transcriptome (scRNA-seq) and immune repertoire (e.g., scTCR-seq) from the same cell, enhancing our understanding of disease mechanisms in cancers [3–7] and autoimmune inflammatory diseases [8, 9]. Various single-cell platforms have been developed, including plate-based methods [10–12], emulsion microfluidic systems [13], nanowells [14], and combinatorial indexing [15, 16], each offering distinct advantages in terms of transcript coverage, sensitivity, and throughput. Developing a high-throughput (HT) single-cell sequencing workflow that combines high sensitivity with broad gene coverage is critical for advancing the field.

In the pursuit of concurrent transcriptome and immune repertoire profiling at single cell resolution, the emulsion microfluidic-based 10X Genomics Chromium with 5’ Solution Kit (10X Genomics, Pleasanton, CA) stands out as a widely adopted and increasingly scalable platform [17–19]. However, plate-based full-length scRNA-seq methods, which utilizes the switching mechanism at the 5′ end of the RNA template (SMART) protocol (e.g., Smart-seq2), provide distinct advantages [10, 20, 21]. Despite their labor-intensive nature, limited throughput, and relatively higher costs, full-length scRNA-seq methods detect a greater number of genes [22, 23] and enable the detection of transcript isoforms, allelic variants, single-nucleotide polymorphisms (SNPs), as well as VDJ rearrangements [24–26]. Previous studies have demonstrated that Smart-seq2 provides significantly higher gene detection sensitivity than 10X [12, 22, 27, 28]. For instance, Xiliang et al. conducted a benchmarking study using the same tissue samples, comparing Smart-seq2 and 10X Chromium [23]. While 10X captured greater cellular heterogeneity attributable to its higher throughput, Smart-seq2 detected more genes [23]. Overall, plate-based SMART methods are well recognized for their superior gene detection sensitivity compared to emulsion microfluidic platforms like 10X, providing more comprehensive transcriptomic data.

Several advancements have improved the scalability and applicability of plate-based SMART protocols for full-length scRNA-seq [20, 21, 29–31]. Lira et al. automated the Smart-seq2 protocol using off-the-shelf reagents combined with robotic implementation, substantially reducing manual labor and experimental costs [29]. Hagemann-Jensen et al. developed the Smart-seq3 [20] and Smart-seq3xpress [21]. Smart-seq3 utilized reduced reagent quantities and incorporated unique molecular identifiers (UMIs) to enhance transcript quantification accuracy. Smart-seq3xpress miniaturized this method, further enhancing its cost-effectiveness and scalability. More recently, Hahaut et al. introduced FLASH-seq which further optimized the cDNA generation process, offering faster turnaround time while maintaining high sensitivity [30]. These advancements, combined with automation and miniaturization, have significantly increased the scalability and efficiency of full-length scRNA-seq, enabling large-scale single-cell studies.

In this study, we built upon the Smart-seq3 protocol [20, 32] to develop the high-throughput Smart-seq3 (HT Smart-seq3) workflow, an automated workflow with a detailed and optimized protocol. Next, to thoroughly assess the performance of HT Smart-seq3 in concurrent single-cell transcriptome and immune repertoire profiling, we profiled human primary CD4 + T-cells and compared HT Smart-seq3 to 10X scRNA/TCR-seq across several key metrics, including gene detection sensitivity, cellular heterogeneity capture, detection of differentially expressed genes, and identification of productive TRA/TRB pairs. Notably, this study represents the first direct comparison of scTCR profiling using the same samples between HT Smart-seq3 and 10X platforms. Through TCR reconstruction, HT Smart-seq3 identified a greater number of productive TRA/TRB pairs. Taken together, our results demonstrate that HT Smart-seq3 workflow produces high-quality data with superior cell capture efficiency and gene detection sensitivity, making it as a promising solution, particularly for low-input, low-RNA content samples.

## Results

### Automated HT Smart-seq3 workflow with an optimized protocol enhances efficiency, scalability, and data quality

The published Smart-seq3 protocol consists of several key steps, starting with single cell collection via FACS, cell lysis, reverse transcription, and cDNA amplification, followed by library generation and sequencing [20, 32]. The primary technical hurdle lies in conducting multiple reactions in individual wells with small reagent volumes, which can introduce manual handling errors and variability. Through hands-on experience with this protocol [32], we also identified the commonly encountered issues, such as inconsistent cell capture rates and failures in library generation, even when cDNA quantification results appeared satisfactory. To tackle these technical challenges, we developed an automated HT Smart-seq3 workflow, incorporating best practices and optimizing the protocol to enhance work efficiency and method reproducibility, ultimately delivering high-quality data.

**Fig. 1:**
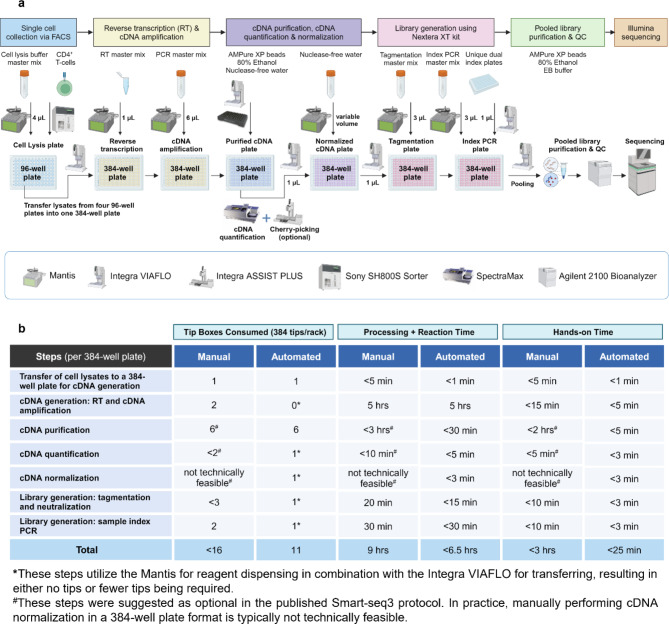
Development of an automated HT Smart-seq3 workflow enhances throughput and efficiency. **a** Schematic view of the streamlined HT Smart-seq3 workflow, illustrating the liquid handling systems (Mantis, Integra VIAFLO 96, and Integra ASSIST PLUS) utilized at each stage. Arrows indicate the volumes dispensed during key steps of the process. **b** Comparison of consumption and time per 384-well plate for each key step following cell lysis between manual Smart-seq3 and automated HT Smart-seq3, highlighting the substantial reduction in hands-on time achieved by automation

In this study, we present a detailed robotic implementation of the HT Smart-seq3 workflow, utilizing readily available reagents and tailored for a 384-well plate format, achieved through the seamlessly integration of several benchtop liquid handling systems (Fig. 1a). Specifically, we combined the Mantis and Integra VIAFLO to handle each step, allowing for efficient and precise processing of multiple 384-well plate in parallel (Fig. 1a). The throughput of this workflow is primarily determined by the number of available thermocyclers. For instance, with six thermocyclers, up to six 384-well plates can be processed in a single batch, which equates to over 2,000 cells. Additionally, compared to the manual Smart-seq3, our automated HT Smart-seq3 reduced tip box consumption and significantly decreased hands-on time, enabling faster processing while maintaining the same reaction time (Fig. 1b).

At the stage of FACS-based cell collection, we frequently encountered challenges with low or varied well occupancy rates when using 384-well plates, likely due to reagent evaporation during prolonged sorting procedures. In this study, we addressed this by switching to 96-well plates for cell collection (Fig. 1a), which significantly reduces sorting time from approximately 8 min per 384-well plate to about 2 min per 96-well plate. This adjustment consistently resulted in a high percentage of wells containing cells, typically achieving over 95% well occupancy per 96-well plate. Following cell lysis, we combined four 96-well plates into a single 384-well plate to streamline downstream reactions (Fig. 1a). Given that sorting time and evaporation rates can vary among different FACS instruments and environmental factors (e.g., hood placement, ventilation, temperature), our approach may not be universally applicable. However, sorting cells into 96-well plates offers a practical solution, particularly for collecting rare cell populations that require extended sorting times, as it minimizes evaporation and ensures optimal well occupancy. The recently published Smart-seq3xpress protocol also addresses evaporation effectively through the use of a hydrophobic overlay [21].

While several established plate-based SMART protocols suggest that certain time-consuming and labor-intensive steps like cDNA purification, quantification, and normalization can be optional [20, 21, 30, 32], we found these steps essential and incorporated them into our HT Smart-seq3 workflow prior to library generation (Fig. 1a) for the following reasons.

First and foremost, cDNA quantification serves as a critical early quality control step in our workflow, allowing us to assess well occupancy following cell collection via FACS. If cDNA quantification reveals a low percentage of wells containing cells, due to issues such as cell collection failures or problems with cDNA generation, we can halt the workflow before advancing to more resource-intensive steps like library generation and sequencing. This early intervention is crucial, given that the estimated cost per cell of our optimized HT Smart-seq3 protocol is approximately $0.50 for cDNA generation, $7.50 for library generation, and $7.50 for sequencing. Additionally, to enable cDNA quantification for larger sample sizes involving thousands of cells, we modified the Qubit assay by using reduced reagent volumes and measured with the SpectraMax fluorescence microplate reader in a 384-well plate format (Fig. 1a). This modification significantly reduced the cost per 384-well plate from approximately $120 to $20. Collectively, using cDNA quantification as an early gating strategy not only saves unnecessary efforts and resources but also, more importantly, prevents the generation of poor-quality data.

Following satisfactory cDNA quantification, cDNA normalization is a vital step to ensure consistent input for library generation. Manual normalization of large sample sizes, based on cDNA quantification results, is often challenging and error-prone. To address this, we employed the Mantis to precisely dispense calculated volumes of diluent into each well, a task typically completed within two minutes per 384-well plate (Fig. 1a). The Integra VIAFLO was then utilized to simultaneously transfer and mix 1 µL of cDNA into the diluent, normalizing cDNA concentrations of 100 pg/µL across all samples (Fig. 1a). Using this uniform input for library generation minimized variability, enabling more reliable pooled library purification and eliminating the need for library normalization prior to sequencing. Importantly, our sequencing results showed relatively even read distribution across samples, demonstrating the effectiveness of our workflow.

As accurate cDNA quantification is crucial as an early quality control step and necessary for cDNA normalization in our workflow, we found that cDNA purification is essential to achieve this accuracy. Without purification, residual oligos (e.g., primers, primer dimers, or dNTPs) can artificially inflate cDNA concentration measurements, particularly in cell-free wells. As shown in Figure S1 (Additional file S1: Figure S1), prior to purification, both samples from cell-containing and cell-free wells exhibited inflated cDNA yields of 78 ng and 103 ng, respectively, as measured by Qubit. This inflation, confirmed by Bioanalyzer, was due to the presence of shorter residual oligos (< 200 bp) (Additional file S1: Figure S1). After purification, the cell-containing well showed a corrected cDNA yield of 70 ng, with fragments ranging from 400 to 9,000 bp, whereas the cell-free well showed negligible cDNA with nearly zero yield by Qubit (Additional file S1: Figure S1). These results clearly underscore the necessity of cDNA purification for obtaining accurate quantification and preventing false-positive signals from cell-free wells. Additionally, to enable efficient cDNA purification, we used pre-programmed Integra VIAFLO to automate this process for an entire 384-well plate (Fig. 1a). When handling multiple plates in a single batch, we overlap steps such as bead addition and incubation, significantly reducing overall processing time.

One technical challenge we initially encountered with the Smart-seq3 protocol [32] was the failure to generate libraries, despite satisfactory cDNA quantification results. Upon reviewing the Nextera XT DNA Library Preparation Kit manual, we found that the issue was likely due to the relatively small amount of Amplicon Tagmentation Mix (ATM; i.e., Tn5 enzyme mix) used in this protocol [32]. To resolve this, we modified the library preparation protocol by increasing the usage of ATM as described in the Materials and Methods section and our protocols.io page (https://www.protocols.io/view/high-throughput-smart-seq3-ewov196jolr2/v1*).* Although this adjustment raised the cost per cell due to the increased ATM usage, it consistently led to successful library generation and thus improved method reproducibility.

Finally, to address occasional variability in well occupancy across multiple plates during FACS-based cell collection, we introduced the Integra Assist Plus system as an optional solution (Fig. 1a). It can selectively pick wells containing cells based on cDNA quantification results. By consolidating the wells with cells into 384-well plates for library generation, we avoid the unnecessary processing of empty wells. This approach further emphasizes the importance of rigorous cDNA quantification in ensuring reliable results and high-quality data.

Taken together, our automated HT Smart-seq3 workflow with established best practices and an optimized protocol enhances efficiency, scalability, and method reproducibility, ultimately ensuring high-quality data.

### Quality assessment of HT Smart-seq3 by comparison to 10X platform

**Fig. 2:**
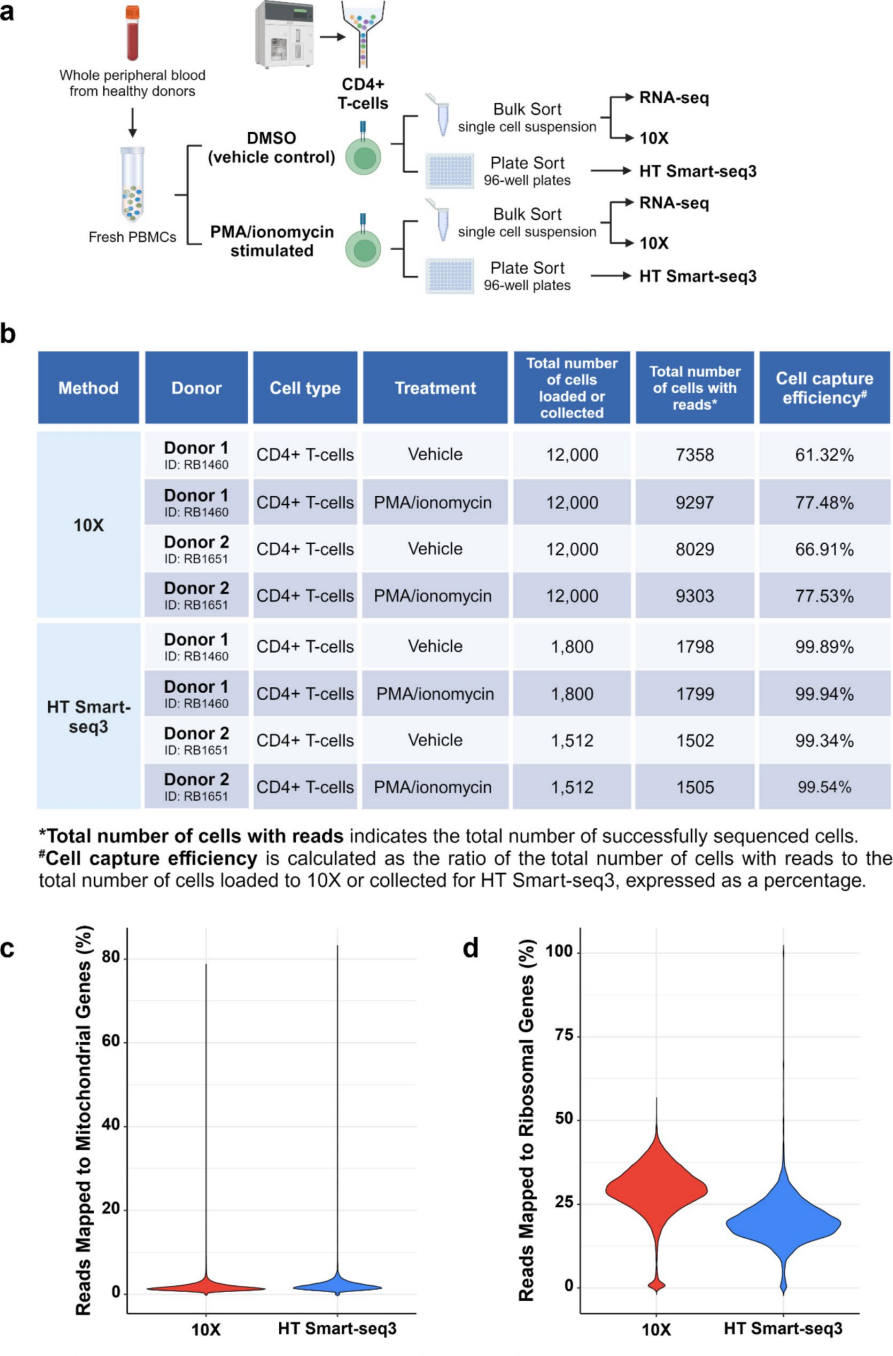
Overview of experimental design and quality assessment. **a** Schematic view of experimental design. **b** Cell capture efficiency of 10X and HT Smart-seq3. **c** Percentage of reads mapped to mitochondrial genes. **d** Percentage of reads mapped to ribosomal genes

To thoroughly evaluate the performance of our HT Smart-seq3 workflow for simultaneous single-cell profiling of transcriptome and immune repertoire, we applied it to profile human primary CD4 + T-cells, which are characterized by low RNA content, and compared the results with 10X scRNA/TCR-seq data generated from the same samples. Here, we used two healthy donors in our study. As shown in Fig. 2a, human peripheral blood mononuclear cells (PBMCs) were isolated from each donor, followed by treatment of DMSO (vehicle control) or PMA/ionomycin. After a 3-hour incubation, CD4 + T-cells were collected via FACS and profiled using either 10X scRNA/TCR-seq or HT Smart-seq3 (Fig. 2a). Bulk RNA-seq data were also generated for all these samples (Fig. 2a).

First, we examined the cell capture efficiency from both the 10X and HT Smart-seq3 platforms. Specifically, cell capture efficiency is calculated as the ratio of the total number of successfully sequenced cells (as shown in the column of “Total number of cells with reads” in Fig. 2b) to the total number of cells either loaded to 10X or collected for HT Smart-seq3 (as shown in the column of “Total number of cells collected” in Fig. 2b), expressed as a percentage. 10X exhibited a capture efficiency ranging from approximately 60% to close to 80% (Fig. 2b), consistent with the expected efficiency for the 10X Chromium platform [33]. In contrast, HT Smart-seq3 demonstrated exceptional capture efficiency with majority surpassing 99% (Fig. 2b). These results highlight the effectiveness of our refined cell sorting procedure using 96-well plates, which consistently achieved high cell capture efficiency across samples. More importantly, it suggests that HT Smart-seq3 is particularly well-suited for samples with a limited cell number of interest.

Next, we evaluated experimental quality by analyzing the proportion of reads mapped to the mitochondrial transcripts, as elevated levels of mitochondrial reads often indicate poor cell quality and ambient RNA contamination [34, 35]. Our results showed that the vast majority (> 97%) of cells from both 10X and HT Smart-seq3 datasets contained less than 5% of reads mapped to mitochondrial genes (Fig. 2c), indicating high-quality cells across both platforms. Furthermore, we examined the proportion of reads mapped to ribosomal protein transcripts, including both large (RPL) and small (RPS) subunits, as excessive mapping to these transcripts can reduce sensitivity in detecting low-abundance genes by consuming sequencing capacity. Our results revealed that HT Smart-seq3 (median % = 19.69%) exhibits a lower proportion of reads mapped to ribosomal genes compared to 10X (median % = 29.80%) (Fig. 2d), suggesting that HT Smart-seq3 may offer enhanced sensitivity in gene detection.

Finally, for our HT Smart-seq3 dataset, we analyzed the gene body coverage for both internal and 5′ UMI-containing reads using 160 randomly selected cells from each sample, representing 10% of the total cell count. As expected, internal reads displayed typical full-length gene body coverage (Additional file S1: Figure S2a), while 5′ UMI-containing reads showed concentrated coverage at the 5′ end (Additional file S1: Figure S2b). The median fraction of 5′ UMI-containing reads was approximately 0.4 across the four samples analyzed in this study (Additional file S1: Figure S2c).

To ensure reliable downstream analysis, we filtered out low-quality cells by excluding those with more than 5% of reads mapped to mitochondrial genes and those with fewer than 200 detected genes. We then employed Celltypist [36] for automated cell type annotation and removed cells not classified as T-cells. As a result, we retained 33,234 cells (97.78% of the 33,987 cells with reads shown in Fig. 2b) from the 10X dataset and 6,415 cells (97.14% of the 6,604 cells with reads shown in Fig. 2b) from the HT Smart-seq3 dataset for downstream analyses.

### HT Smart-seq3 demonstrates higher sensitivity in gene detection and a reduced dropout rate

**Fig. 3:**
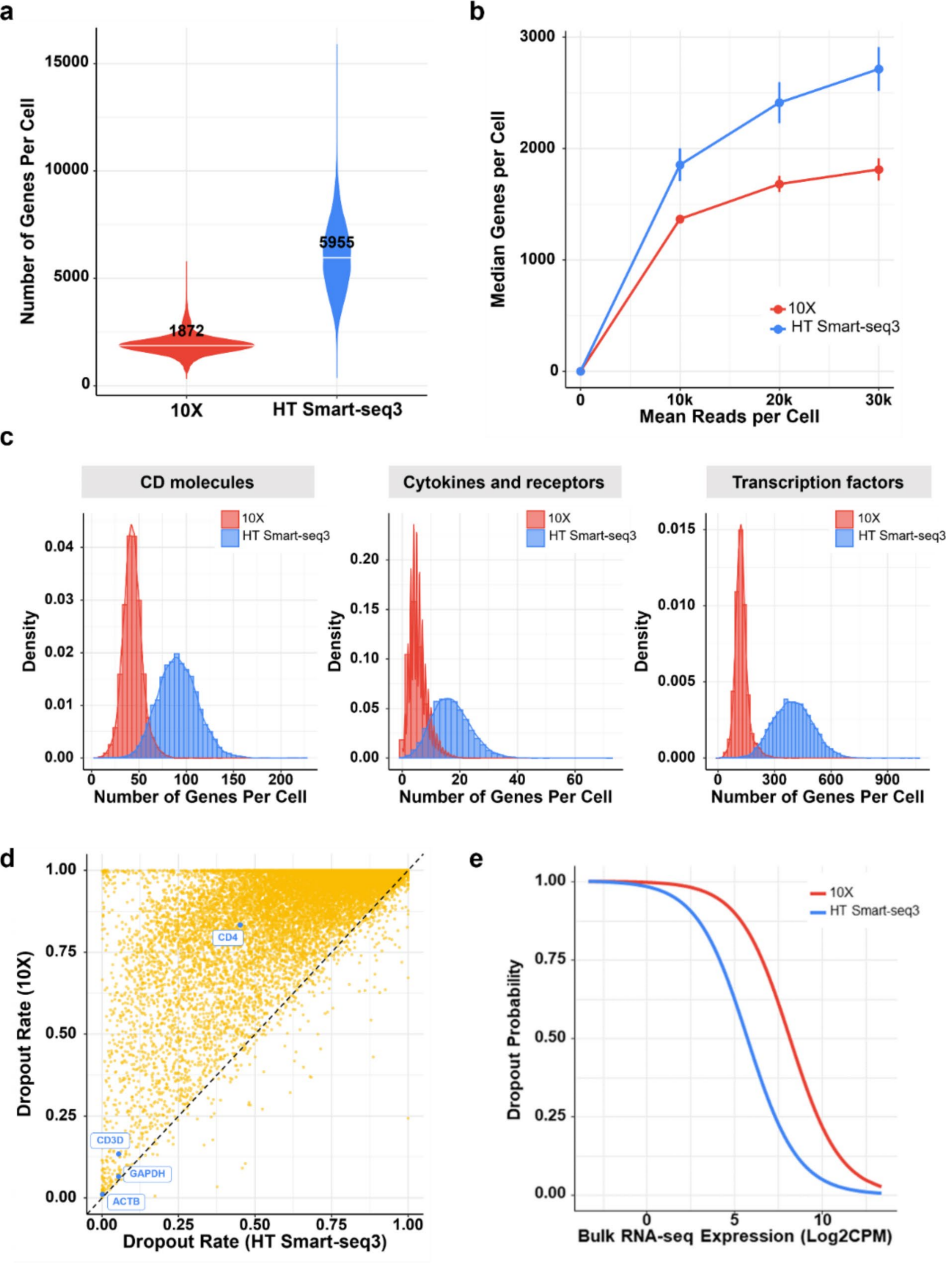
HT Smart-seq3 detects more genes and exhibits lower dropout rate. **a** Comparison of gene detection sensitivity between 10X and HT Smart-seq3. **b** Sequencing depth-normalized comparison of gene detection sensitivity between 10X and HT Smart-seq3. **c** Distribution of the number of detected genes in various gene categories, including CD molecules, cytokines and their receptors, as well as transcription factors. **d** Comparison of dropout rates between 10X and Smart-seq3. **e** Relationship of dropout rates with gene expression levels in bulk RNA sequencing data

To assess whether the dataset generated by our HT Smart-seq3 workflow retains superior gene detection sensitivity compared to 10X, we compared the number of genes detected per cell between these two platforms. For HT Smart-seq3 data, all reads (i.e., both internal and 5’ UMI-containing reads) were used, and a gene was considered detected if at least one uniquely mapped read was assigned to it. Our results revealed that HT Smart-seq3 exhibited significantly higher sensitivity, detecting a median of 5,955 genes per cell, compared to 1,872 genes per cell detected by 10X (Fig. 3a). To address the potential influence of sequencing depth on this observation, we down-sampled the sequencing reads from HT Smart-seq3 to match the depth of 10X. Remarkably, even after this adjustment, HT Smart-seq3 consistently outperformed 10X in detecting a greater number of genes per cell (Fig. 3b), reaffirming its superior gene detection sensitivity.

To gain a deeper understanding of the biological relevance of the broader gene spectrum detected by HT Smart-seq3, we focused on specific categories, including CD molecules, cytokines and their receptors, and transcription factors, given the fact that deregulation of genes within these categories can impact T-cell functions [37–39]. Our analyses demonstrated that HT Smart-seq3, compared to 10X, consistently detected two to three times more genes across these categories (Fig. 3c), underscoring its sensitivity and potential for uncovering more profound biological insights.

Dropout events in scRNA-seq occur when certain genes fail to be detected, leading to an overabundance of expression values set to zero. This phenomenon poses challenges in reliably identifying differentially expressed genes [11, 40]. As the 10X platform is known for its relatively higher dropout rates, we assessed dropout rates for HT Smart-seq3. Specifically, the dropout rate for each gene was determined by calculating the proportion of cells with zero expression values for that gene relative to the total number of cells in the dataset. Our results revealed that HT Smart-seq3 exhibited lower dropout rates for most genes compared to 10X (Fig. 3d), while the dropout rates of housekeeping genes (e.g., GAPDH, ACTB) were comparable (Fig. 3d). For instance, the dropout rate for CD3D gene (a T-cell surface maker) was 7.7% in HT Smart-seq3 and 15.0% in 10X (Fig. 3d). Similarly, the dropout rate for CD4 gene (a surface marker of helper T-cells) was 43.6% in HT Smart-seq3 and 83.4% in 10X (Fig. 3d).

In addition to scRNA-seq datasets, we generated bulk RNA-seq data from the same samples to investigate the relationship between dropout rates and gene expression levels derived from bulk RNA-seq for each single-cell platform. By fitting a modified non-linear Michaelis-Menten model [41], our analyses indicated that, as anticipated, genes with lower expression levels tend to exhibit higher dropout rates; additionally, HT Smart-seq3 consistently demonstrated lower dropout rates compared to 10X (Fig. 3e).

Taken together, coupled with its enhanced gene detection sensitivity and lower dropout rates, HT Smart-seq3 demonstrated its reliability in capturing the more comprehensive transcriptomic landscape.

### HT Smart-seq3 captures major cellular heterogeneity by scaling up

**Fig. 4:**
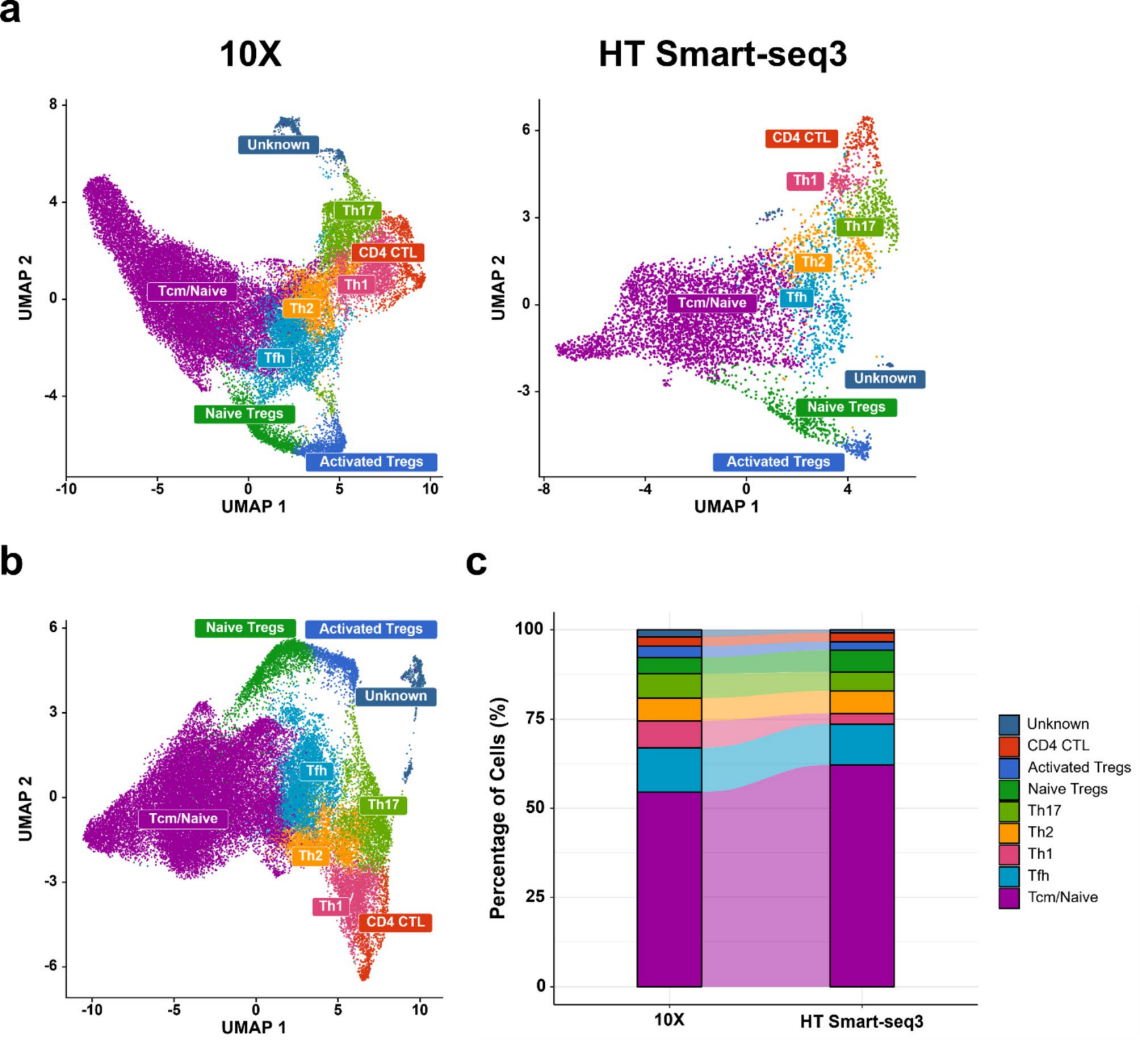
HT Smart-seq3 captures major T-cell subtypes as 10X. **a** UMAP visualization of cells separated by technology. **b** Cell clustering and annotation of the CD4 + T-cell subtypes in the integrated 10X and HT Smart-seq3 data. **c** Comparison of cell type compositions between the 10X and HT Smart-seq3 data

In a previous benchmarking study comparing 10X and Smart-seq2 on tumor and adjacent non-tumor tissues, more than 1,000 cells per sample were profiled by 10X, whereas fewer than 200 cells per sample were profiled by Smart-seq2. Their results showed that 10X identified more cell clusters, likely attributed to its higher throughput [23]. In this study, we profiled over 7,000 cells per sample by 10X and over 1,500 cells per sample by HT Smart-seq3 (Fig. 2b). Independent analysis of the 10X and HT Smart-seq3 datasets showed both platforms were able to effectively capture the major CD4 + T-cell subtypes, including naïve and central memory T-cells (Tcm/Naive), T follicular helper cells (Tfh), Th1, Th2, Th17, regulatory T-cells (Treg), and cytotoxic CD4 + T-cells (CD4 + CTL) (Fig. 4a). These findings indicate that adequately scaling up the HT Smart-seq3 dataset can achieve resolution comparable to that of 10X.

Next, we integrated both 10X and HT Smart-seq3 datasets via canonical correlation analysis (CCA) using the Seurat package [42]. Our integrative analysis also identified the same major CD4 + T-cell subtypes from both 10X and HT Smart-seq3 (Fig. 4b) as the independent analysis. The cell compositions were also consistent between these two platforms (Fig. 4c and Additional file S2: Table S1). Additionally, no discernible batch effects were observed from either the platforms or the donors (Additional file S1: Figure S3). Taken together, our study underscores the importance of the throughput that can be achieved by scRNA-seq platforms. Therefore, the scalability of HT Smart-seq3 makes it a promising platform, offering comparable performance to 10X for capturing cellular heterogeneity and accurately identifying cell subsets.

### HT Smart-seq3 demonstrates enhanced detection of differentially expressed genes (DEGs)

Differential gene expression analysis in scRNA-seq data enables the identification of genes with varying expression levels between distinct biological conditions for each specific cell subtype. In this study, we performed differential gene expression analyses comparing PMA/ionomycin to vehicle treatment for each T-cell subtype identified from both 10X and HT Smart-seq3 datasets.

**Fig. 5:**
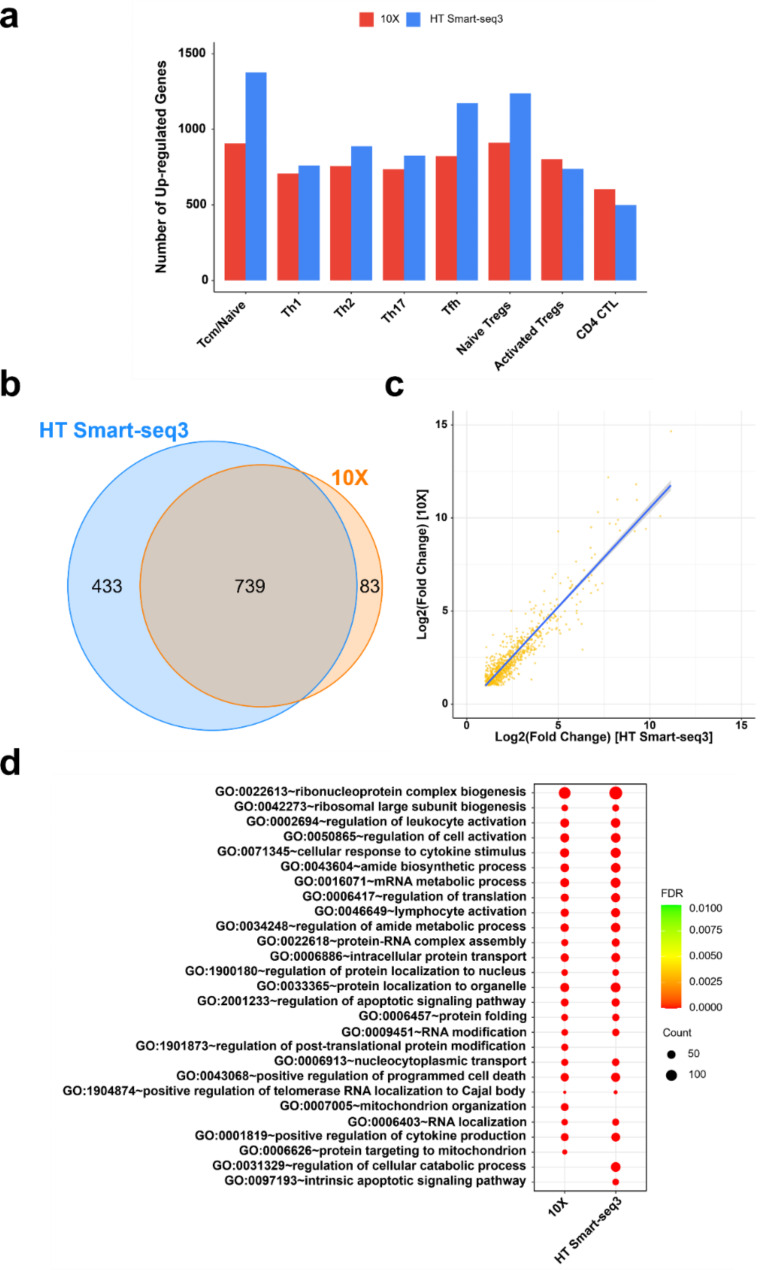
Comparison of DEGs and pathways upon PMA/ionomycin stimulation between 10X and HT Smart-seq3 datasets. **a** Number of up-regulated DEGs identified in each T-cell subtype upon PMA/ionomycin stimulation. **b** Overlap of up-regulated DEGs in Tfh cells between two platforms. **c** Correlation of fold-change values for commonly up-regulated DEGs in Tfh cells between two platforms. **d** Dysregulated pathways in Tfh cells upon PMA/ionomycin stimulation

Our results revealed that HT Smart-seq3 detected a greater number of DEGs for each major T-cell subtype, despite having fewer cells than the 10X dataset (Fig. 5a). For example, HT Smart-seq3 identified 1,375 up-regulated genes in Tcm/Naïve T-cells following PMA/ionomycin stimulation, whereas 10X identified only 906 up-regulated genes in the same T-cell subtype (Fig. 5a). Known positive controls, such as TNF-alpha, IFN-gamma, IL-4, and IL-10, which are upregulated upon PMA/ionomycin stimulation [43, 44], were significantly upregulated in specific T-cell subtypes in both 10X and HT Smart-seq3 data (Additional file S1: Figure S4a). These findings were also validated by bulk RNA-seq generated from the same samples (Additional file S1: Figure S4b).

Next, we compared the DEGs between 10X and HT Smart-seq3 datasets, and found a significant overlap between these two platforms. For instance, in Tfh cells, nearly 90% of the up-regulated genes identified in the 10X dataset (739 out of 822 genes, representing 89.9%) were also detected in the HT Smart-seq3 dataset (Fig. 5b). HT Smart-seq3 identified additional 433 up-regulated genes that were not detected in 10X (Fig. 5b), indicating its greater sensitivity in detecting DEGs. Additionally, for the commonly up-regulated genes identified in both datasets, fold-change values were highly correlated (Pearson’s correlation coefficient = 0.93, p-value < 2.2e-16) (Fig. 5c). Lastly, both datasets identified similar up-regulated pathways upon PMA/ionomycin stimulation in Tfh cells (Fig. 5d).

While our results show that HT Smart-seq3 achieves a higher number of DEGs across most T-cell subtypes (Fig. 5a), it is important to note that the number of cells profiled within each condition may influence this result. For instance, activated Tregs identified from HT Smart-seq3 data comprised only 76 cells under PMA/ionomycin stimulation and 124 cells under Vehicle control (Additional file S2: Table S1), resulting in a slightly lower number of up-regulated genes compared to the 10X data (Fig. 5a), although the discrepancy remained comparable. Similarly, fewer DEGs were identified for another small cluster (CD4 + CTL) (Fig. 5a), which comprised less than 80 cells in each condition in the HT Smart-seq3 data (Additional file S2: Table S1). Importantly, upon down-sampling the 10X data to match the cell count of HT Smart-seq3 for each T-cell subtype under every condition, HT Smart-seq3 consistently detected a greater number of DEGs than 10X (Additional file S1: Figure S5). Taken together, it again underscores the critical role of throughput provided by single-cell platforms to ensure robust downstream analysis, highlighting the value of the HT Smart-seq3 workflow.

### HT Smart-seq3 detects a higher proportion of cells with productive TRA and TRB pairs via TCR reconstruction

**Fig. 6:**
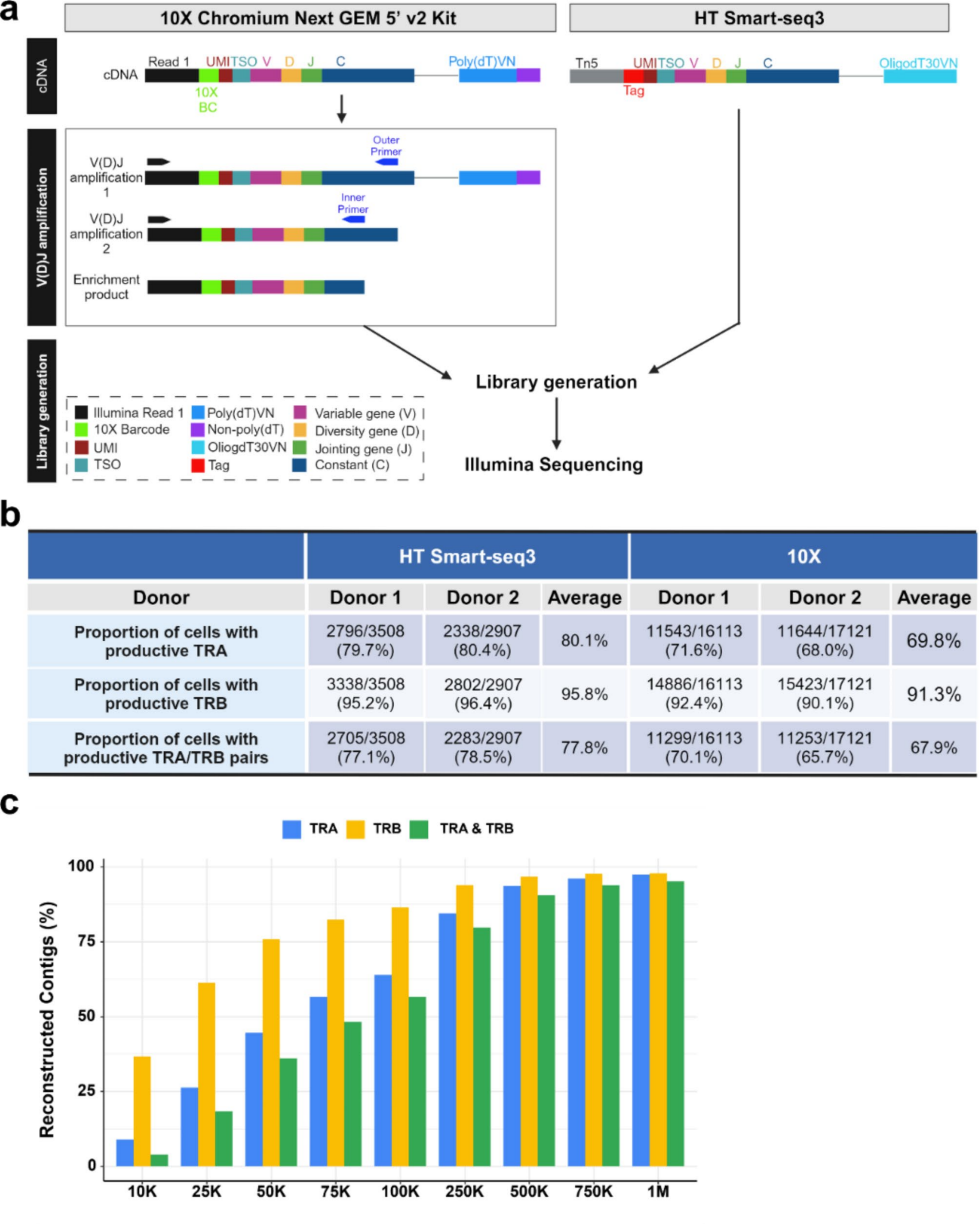
Comparison of scTCR-seq between 10X and HT Smart-seq3. **a** Schematic view of scTCR-seq between two platforms. **b** Proportion of cells with productive TRA and TRB identified from 10X scTCR-seq and HT Smart-seq3 datasets. **c** Percentage of reconstructed TRA/TRB contigs matching the “ground-truth” (i.e., contigs assembled using all available reads) at various down-sampled sequencing depths in the HT Smart-seq3 dataset

The main advantage of scTCR-seq is its ability to provide paired TRA and TRB information, allowing for precise identification of T-cell clonotypes and their corresponding antigen specificity [1, 2]. The widely adopted 10X scTCR-seq involves generating an additional TCR library by PCR amplification of V(D)J segments from full-length cDNAs using species-specific primer sets (Fig. 6a). This approach may introduce PCR amplification bias, potentially leading to less accurate quantification and reduced sensitivity, especially in detecting low-frequency TCRs [43]. Alternatively, full-length scRNA-seq, like Smart-seq3, enables concurrent transcriptome profiling and immune receptor repertoire reconstruction from a single transcriptome library through TCR reconstruction (Fig. 6a), potentially offering a more accurate and sensitive approach for TCR repertoire identification. However, to date, no study has directly compared these two platforms for scTCR-seq.

To fill this gap, we conducted 10X scTCR-seq and HT Smart-seq3 on the same samples of primary human CD4 + T-cells. Specifically, we employed TraCeR to assemble reads into TCR contigs for each cell from the HT Smart-seq3 dataset [21], and subsequently compared the results with the 10X scTCR-seq dataset. Our findings revealed that, across both platforms, a higher proportion of cells were identified with productive TRB, 91.3% from 10X and 95.8% from HT Smart-seq3, compared to TRA, 69.8% from 10X and 80.1% from HT Smart-seq3 (Fig. 6b); these findings indicate that HT Smart-seq3 detected approximately 10% more TRA and TRB than 10X. Furthermore, 77.8% of cells from HT Smart-seq3 datasets exhibited productive TRA and TRB pairs, a notable improvement compared to 67.9% achieved from 10X (Fig. 6b). These results were consistent between the two donors (Fig. 6b). It should be noted that all productive TRA/TRB contigs reported in the study have annotated CDR3 sequences. Additionally, a down-sampling analysis for HT Smart-seq3 data was performed to investigate the sequencing depth that is required to reconstruct the same TRA/TRB contigs as the “ground-truth” (i.e., those assembled using all available reads). As shown in Fig. 6c, at a sequencing depth of 250 K reads, 84.4% of cells had the same TRA sequences reconstructed, 94.4% for TRB, and 79.7% for both TRA and TRB pairs. Increasing the sequencing depth to 500 K reads further improved the reconstruction of TRA and TRB pairs to 90.5% of cells (Fig. 6c).

Finally, we identified consistent TCR clonotypes across both platforms and did not observe clonotype expansion upon PMA/ionomycin treatment (data not shown). No TCR clonotype expansion was observed upon stimulation; this observation is aligned with our expectations, considering naïve T-cells require prolonged antigenic stimulation and our use of CD4 + T-cells isolated from healthy donors with only a 3-hour stimulation [44]. Further investigation is warranted to profile samples with anticipated TCR clonotype expansion, such as antigen-experienced or disease-specific T-cells.

## Discussion

Pathogenic T-cell subsets, such as CD4 + T-cells, play pivotal roles in a variety of diseases, including cancers [3–7] and autoimmune inflammatory conditions [8, 9]. TCRs, composed of alpha (TRA) and beta (TRB) chains, are responsible for recognizing and responding to diverse antigens to mediate immune responses [36]. Understanding the diversity of the TCR repertoire and the clonal expansion of T-cells across different states, tissues, and time points during disease progression is crucial for unraveling immunological mechanisms and developing targeted therapeutic interventions [45, 46]. Compared to bulk TCR-seq, scTCR-seq offers the unique advantage by capturing individual T-cells along with their paired TRA and TRB information [1, 2], facilitating precise identification of T-cell clonotypes and their antigen specificities. Therefore, concurrent profiling of transcriptome and TCR repertoire at single cell resolution holds immense potential for advancing our understanding of disease mechanisms and therapeutic strategies. However, challenges arise due to the low RNA content and limited cell numbers in many T-cell subsets [33, 47], which can impact the performance of chosen single-cell platforms for scRNA/TCR-seq. Each platform possesses unique limitations and capabilities [12], making the choice of method crucial.

While the emulsion microfluidic-based 10X Chromium platform with its 5’ Solution Kit is widely utilized for scRNA/TCR-seq, plate-based full-length scRNA-seq methods like Smart-seq3 offer distinct advantages for deeply profiling rare cell types or those with low RNA abundance. Despite several efforts in optimizing SMART protocols [20, 21, 29, 30], opportunities remain for further enhance their feasibility and robustness for large-scale, high-resolution cell atlases. To address these challenges, we developed the high-throughput Smart-seq3 (HT Smart-seq3) workflow, which automates key steps using liquid handling systems and incorporates best practices with an optimized protocol. This approach overcomes technical barriers in throughput and reproducibility, resulting in superior performance and high-quality data. In addition, we conducted a comprehensive assessment of its performance alongside 10X scRNA/TCR-seq by profiling human primary CD4 + T-cells (the cells with low RNA content) isolated from fresh whole blood. This comparison allowed us to elucidate the strengths and limitations of each single-cell platform.

First, leveraging well-established FACS panels for T-cell subset identification, 10X scRNA/TCR-seq requires sorting a larger number of live T-cells of interest as a pool, followed by immediate processing through 10X Chromium platform, typically achieving cell capture rates ranging from 60 to 80% [33] (Fig. 2b). In contrast, the HT Smart-seq3 workflow offers greater flexibility by allowing individual cell sorting into wells that can be stored at -80 °C for later batch processing. More importantly, our optimized protocol, particularly the improved cell sorting procedure, consistently results in a high percentage of wells containing cells, leading to superior cell capture efficiency (Fig. 2b). Additionally, we incorporate an early quality control step via cDNA quantification to identify any issues before library generation (Fig. 1a), avoiding unnecessary resource use and ensuring data integrity. Collectively, these features make HT Smart-seq3 a flexible and effective solution for studies with a limited number of cells of interest.

Second, our results showed that compared to 10X, HT Smart-seq generates high-quality data with greater gene detection sensitivity and reduced dropout rates, particularly for low-expressing genes. Importantly, despite 10X’s reputation for identifying rare cell subsets, presumably achieved by its higher throughput [23], HT Smart-seq3 demonstrated comparable capabilities in capturing diverse major T-cell subtypes when sufficiently scaled (Fig. 4). Additionally, HT Smart-seq3 consistently outperformed 10X in detecting differentially expressed genes following PMA/ionomycin stimulation (Fig. 5). Taken together, our findings suggest that HT Smart-seq3 is particularly valuable for low-input or low-expression samples, such as pathogenic T-cells from diseased tissues.

Finally, HT Smart-seq3 demonstrated superior performance in TCR detection, as evidenced by its ability to reconstruct more productive TRA/TRB pairs (Fig. 6b). One key distinction between 10X and HT Smart-seq3 lies in their library generation process (Fig. 6a). 10X requires generating two separate libraries: one for gene expression through 5’ tag sequencing and another for TCR through full-length V(D)J sequencing. This process necessitates additional PCR amplification of full-length V(D)J segments from amplified cDNA using species-specific primer sets, which can introduce concerns about primer specificity and amplification bias due to differential primer efficiency [43, 48]. In contrast, HT Smart-seq3 requires only a single library, as TCR information can be reconstructed directly from full-length transcripts. This eliminates potential errors associated with primer design and reduces amplification bias, offering a distinct advantage in TCR profiling. By avoiding the use of species-specific primers, HT Smart-seq3 also expands applicability across a broader range of species beyond human and mouse. Future studies are planned to utilize HT Smart-seq3’s capacity for tracking TCR repertoire diversity and clonal expansion in antigen-experienced or disease-specific T-cells.

## Conclusion

In this study, we developed an automated HT Smart-seq3 workflow that incorporates best practices and an optimized protocol to enhance efficiency, scalability, and method reproducibility, resulting in high-quality data with superior gene detection sensitivity. Through rigorous comparison with 10X scRNA/TCR-seq on primary CD4 + T-cells, HT Smart-seq3 outperformed 10X by exhibiting higher cell capture efficiency, greater gene detection sensitivity, and lower dropout rates. When sufficiently scaled, HT Smart-seq3 achieved the comparable resolution of cellular heterogeneity as 10X. Furthermore, without the need for additional primer design to amplify full-length V(D)J segments, HT Smart-seq3 detected more productive TRA/TRB pairs via TCR reconstruction, enabling more unbiased scTCR profiling across a wider range of species. Taken together, these technical strengths position HT Smart-seq3 as a promising solution, particularly well-suited for low-input, low RNA content samples.

## Materials and methods

### PBMC preparation, treatment and CD4 + T-cell sorting

Peripheral whole blood was collected from two healthy donors (Donor 1, ID RB1460; Donor 2, ID RB1651) in ethylenediaminetetraacetic acid (EDTA)-coated tubes by BioIVT (Colmar, PA, USA) [49]. Upon receipt, peripheral blood mononuclear cells (PBMCs) were isolated using SepMate tubes (STEMCELL Technologies, Cat. #85450) following the manufacturer’s instructions. The isolated PBMCs were resuspended in RPMI 1640 medium (Gibco, Cat. #11875093) supplemented with 5% human serum (Sigma, Cat. #H3667-100ML) and 1% penicillin-streptomycin. After overnight incubation, PBMCs were resuspended and divided into two pools: one treated with DMSO (vehicle control) and the other with with phorbol-12-myristate-13-acetate plus ionomycin (PMA/ionomycin) cell stimulation cocktail (eBioscience, Cat. #00-4970) for 3 h. The PBMCs were then incubated with eFluor 780 viability dye (Invitrogen, Cat. #65-0865-14) to exclude dead cells, and stained with APC-conjugated mouse anti-human CD4 antibody (SK3, BD Biosciences, Cat. #340443) and AlexaFluor488-conjugated mouse anti-human CD3 antibody (UCHT1, BioLegend, Cat. #300454). CD4 + T-cells were sorted via FACS using SH800S cell sorter (Sony Biotechnology Inc, San Jose, CA, USA) equipped with a 100-µm nozzle. For 10X and bulk RNA-seq, live CD4 + T-cells were sorted into tubes containing PBS with 1% BSA. For HT Smart-seq3, live single CD4 + T-cells were sorted into individual wells of 96-well plates containing cell lysis buffer. These plates were immediately stored at − 80 °C until further processing.

### High-throughput Smart-seq3

We developed the automated HT Smart-seq3 workflow (Fig. 1a) by integrating multiple benchtop liquid handling systems, incorporating best practices, and optimizing the protocol based on the published Smart-seq3 method [20, 32]. A detailed version of our HT Smart-seq3 protocol is available on protocols.io (https://www.protocols.io/view/high-throughput-smart-seq3-ewov196jolr2/v1).

#### Single cell collection

Prior to cell sorting, 4 µL of cell lysis buffer (containing 5% PEG8000 (Sigma), 0.1% Triton X-100 (Sigma), 0.5 U/µl of RNase inhibitor (Takara), 0.5 µM Smart-seq3 oligo-dT-30VN (Integrated DNA Technologies), and 0.5 mM/each dNTP (Thermo Fisher Scientific)) was dispensed into 96-well plates using Mantis (Formulatrix, Bedford, MA, USA). After single cell collection via FACS, the plates were then stored at -80 °C for batch processing at a later time.

#### Cell lysis, reverse transcription, and cDNA amplification

These 96-well plates were incubated at 72 °C for 10 min to lyse and denature the cells. To prepare for reverse transcription (RT), 1 µL of RT master mix (containing 25 mM Tris-HCl pH 8.3 (Sigma), 30 mM NaCl (Invitrogen), 2.5 mM MgCl₂ (Invitrogen), 1 mM GTP (Thermo Fisher Scientific), 8 mM DTT (Thermo Fisher Scientific), 0.5 U/µL RNase inhibitor (Takara), 2 µM Smart-seq3 TSO (Integrated DNA Technologies), and 2 U/µL Maxima H-minus reverse transcriptase (Thermo Fisher Scientific)) was dispensed into 384-well plates using Mantis. The cell lysates from the 96-well plates were then transferred and combined into the 384-well plates pre-filled with RT master mix using Integra VIAFLO (INTEGRA Biosciences, Tokyo, Japan). RT was performed as follows: incubation at 42 °C for 1 h and 30 min, followed by 10 cycles of incubation at 50 °C for 2 min and 42 °C for 2 min, then incubation at 85 °C for 5 min, followed by holding at 4 °C. After completing RT, 6 µL of PCR master mix (containing 1X KAPA HiFi HotStart PCR buffer (Roche), 0.02 U/µl DNA polymerase (Roche), 0.3 mM/each dNTPs, 0.5 mM MgCl_2_ (Invitrogen), 0.5 µM Smart-seq3 forward primer (Integrated DNA Technologies), and 0.1 µM Smart-seq3 reverse primer (Integrated DNA Technologies)) was added into the same 384-well plates using Mantis to carry out cDNA amplification. For CD4 + T-cells used in this study, cDNA amplification was performed with an initial denaturation at 98 °C for 3 min, followed by 25 cycles of 98 °C for 20 s (denaturation), 65 °C for 30 s (annealing), and 72 °C for 4 min (elongation), concluding with a final elongation at 72 °C for 5 min and a hold at 4 °C.

#### cDNA purification

Automated cDNA purification was performed using Integra VIAFLO with a custom program designed specifically for beads clean-up. In brief, 6 µL of well-mixed AMPure XP beads (Beckman Coulter, Cat. #A63882), equivalent to 0.6X the reaction volume, were added to each well of the 384-well plate containing the amplified cDNA. The cDNA and beads were thoroughly mixed by pipetting and then incubated at room temperature for 5 min. After incubation, the plate was placed on a 384-well ring magnetic plate (Permagen, Cat. #MSP384R) for efficient magnetic bead separation. The supernatant was then carefully removed, and the beads were washed twice with 30 µL of 80% ethanol for 30 s each. The beads were air-dried at room temperature for 2 min to remove any residual ethanol. Finally, purified cDNA was eluted in 10 µL of nuclease-free water.

#### cDNA quantification and normalization

Upon completion of cDNA purification, cDNA quantification was performed using Qubit dsDNA High Sensitive (HS) Assay Kit (Invitrogen, Cat. #Q32854) and the measurement was carried out using SpectraMax microplate reader (Molecular Devices, San Jose, CA, USA). Notably, we implemented a protocol that reduced reagent consumption by more than 80% while maintaining accuracy. In brief, calibration standards were prepared by serial dilution to cover a linear range of 0–5 ng/µL of cDNA, and the Qubit dsDNA HS Reagent was diluted 1:200 in Qubit dsDNA HS Buffer to prepare the Qubit working solution. Then, 34 µL of the Qubit working solution was dispensed into each well of a 384-well black flat-bottom microplate (Corning, Cat. #3820), followed by the addition of 1 µL of either the purified cDNA or calibration standard. Fluorescence was measured using the SpectraMax microplate reader (Excitation: 485 nm; Emission: 525 nm), and cDNA concentrations were calculated based on the resulting fluorescence values. Based on cDNA quantification results, cDNA normalization was performed to adjust all samples to a concentration of 100 pg/µL of purified cDNA. Specifically, Mantis was used to dispense the calculated volumes of nuclease-free water required for dilution into each well of a new 384-well plate. Then, 1 µL of purified cDNA from each sample was added using the Integra VIAFLO to ensure accurate normalization.

#### Optimized library generation and pooled library purification

Library generation was performed with modifications based on the published Smart-seq3 protocol [20, 32]. Normalized cDNA was utilized as the input for library generation using Nextera XT DNA Library Preparation Kit (Illumina, Cat. #FC-131-1096), which includes tagmentation and sample index PCR steps. Unique Dual Indexes (UDIs) Sets A-D plates (Illumina, Cat. #20091654, #20091656, #20091658, #20091660) were used for sample indexing. Specifically, tagmentation master mix (containing 2 µL of Tagment DNA Buffer (TD) and 1 µL of Amplicon Tagment Mix (ATM)) was dispensed into new 384-well plates using Mantis, followed by the addition of 1 µL of normalized cDNA (equivalent to 100 pg) using Integra VIAFLO. The mixture was incubated at 55 °C for 5 min. Then, 1 µL of Neutralize Tagment Buffer (NT) was added using Mantis, followed by a 5-minute incubation at room temperature to release the Tn5 transposase from the cDNA. Next, 3 µL of Nextera PCR Master Mix (NPM) was added using Mantis, followed by the addition of 1 µL of each UDI (diluted 1:1 with Resuspension Buffer (RSB)) to each well using the Integra VIAFLO. Sample index PCR was performed with a gap-filling step at 72 °C for 3 min and an initial denaturation at 95 °C for 30 s, followed by 14 cycles of 95 °C for 10 s (denaturation), 55 °C for 30 s (annealing), and 72 °C for 30 s (elongation), concluding with a final elongation at 72 °C for 5 min and a hold at 4 °C. Libraries with different UDIs were then pooled and purified using AMPure XP beads at a 0.6X ratio.

#### Library QC, and sequencing

For quality control, the concentration of the pooled library was measured using Qubit dsDNA HS Assay Kit, and the size distribution was assessed using Agilent 2100 Bioanalyzer with High Sensitivity DNA kit (Agilent Technologies, Cat. #5067 − 4626). The pooled library was then sequenced on Illumina NovaSeq6000 and NextSeq2000 (Illumina, San Diego, CA, USA) with paired-end 150 bp reads per sample.

### 10X scRNA/TCR-seq

Following CD4 + T-cell collection via FACS, cell viability was assessed using ViaStain AOPI Staining Solution (acridine orange/propidium iodide, Thermo Fisher Scientific, Cat. #CS2-0106-5ML). Cells were then processed for scRNA/TCR-seq using 10X Genomics Chromium Next GEM Single Cell 5’ v2 Kit (10X Genomics, Cat. #PN-1000263) along with the TCR Amplification Kit for Human (10X Genomics, Cat. #PN-1000252). The concentration of the libraries was determined by Qubit assay, and the size distribution was assessed using Agilent 2100 Bioanalyzer with High Sensitivity DNA Kit. Sequencing was performed on Illumina NextSeq2000 and NovaSeq6000, following the manufacturer’s instructions outlined in the 10X Genomics User Guide.

### Bulk RNA-seq

Total RNA was extracted using RNeasy Mini Kit (Qiagen, Cat. #74104), following the manufacturer’s instructions. Prior to library construction, RNA quantity was measured using Qubit RNA HS Assay Kit (Invitrogen, Cat# Q32855), and RNA integrity was assessed using Agilent 2200 TapeStation with High Sensitivity RNA ScreenTape (Agilent Technologies, Cat. #5067–5592). All samples had RIN values above 7, indicating high RNA quality. Approximately 500 ng of total RNA was used for library preparation using TruSeq Stranded mRNA Library Prep Kit (Illumina, Cat# 20020595), following the manufacturer’s instructions. The concentration of the libraries was determined by Qubit assay, and the size distribution was confirmed using an Agilent 2100 Bioanalyzer with the DNA 1000 Kit (Agilent Technologies, Cat. #5067 − 1504). The libraries were multiplexed and sequenced on Illumina NextSeq2000 and NovaSeq6000 platforms, generating paired-end 50 bp reads per sample.

### Data processing and analysis

#### Primary processing for single-cell data

A comprehensive and easy-to-use Nextflow workflow was developed to streamline the processing of scRNA-seq data generated by HT Smart-seq3.

Instead of utilizing undemultiplexed FASTQ files, we began by merging demultiplexed FASTQ files from each plate, followed by analysis with the *zUMIs* tool [11] for gene expression quantification. Our customized pipeline (Custom) demonstrated improved gene detection with comparable data processing times, compared to the best practice pipeline (Standard) recommended in the Smart-seq3 V.3 protocol [32] (Additional file S1: Figure S6). Specifically, the *merge_demultiplexed_fastq.R* script provided in the *zUMIs* tool (v2.9.7) was first used to merge demultiplexed FASTQ files for cells on each plate. Then, the *zUMIs.sh* script was used to execute a series of steps, including filtering, mapping, counting, and summarizing, to generate gene expression count matrices and summary statistics. Modifications of source code in the *runfeatureCountFun.R* script within *zUMIs* excluded multi-mappers (countMultiMappingReads = FALSE) and fragments not mapped to both ends (requireBothEndsMapped = TRUE) from quantification. The read count matrix for both exonic and intronic mapping was used for downstream analysis. For TCR reconstruction from our HT Smart-seq3 data, we employed the Docker image of the *TraCeR* tool (v0.6.0) from Docker Hub (https://hub.docker.com/r/teichlab/tracer/*).* The *assemble* module within TraCeR was first used to reconstruct TCR sequences for individual cell, and the *summarise* module was subsequently used to generate the summary statistics and detailed information for all cells. For our 10X data, we employed the *cellranger mutli* pipeline (v7.1.0) to process both gene expression and TCR profiling. Intronic mapped reads were also included in gene expression quantification. The reference index refdata-gex-GRCh38-2020-A, provided within the *cellranger* package, was used for read alignment and quantification. To ensure consistency in benchmarking, we used the same genome reference sequence (genome.fa) and gene annotation information (genes.gtf) provided by *cellranger* for processing HT Smart-seq3 data.

#### Integrative analysis of 10X and HT Smart-seq3 data

We used the R package *Seurat* (v5.0.0) to integrate the 10X and HT Smart-seq3 datasets for downstream analysis. Default parameters were applied unless otherwise specified. Specifically, low-quality cells, defined as those with more than 5% of reads mapped to mitochondrial genes or fewer than 200 detected genes, were first excluded. The data was then normalized using the “LogNormalize” method, and the top 3,000 variable features were selected with the “vst” method. After scaling the data, principal component analysis (PCA) was performed for linear dimensionality reduction. Prior to integrative analysis, the Celltypist tool was employed for automated cell type annotation to exclude cells that were not annotated as T-cells. In this study, we applied the above steps separately to the 10X and HT Smart-seq3 datasets, and then integrated the data using canonical correlation analysis (CCA) method. Subsequent analyses, including cell clustering, uniform manifold approximation and projection (UMAP) for dimension reduction, cell type annotation, and differential gene expression analysis, were performed on this integrated dataset. Cell clustering was conducted using the Leiden algorithm with a resolution of 1.2 in the *FindClusters()* function within Seurat. A Wilcoxon rank sum test was employed to identify marker genes for each cluster, and canonical CD4 + T-cell subtype markers were used to further annotate the cell cluster. Sub-clustering was performed when cell type assignments were ambiguous. Differentially expressed genes (DEGs) for each cell type in response to PMA/ionomycin stimulation were identified using the Wilcoxon rank sum test, with a Benjamini-Hochberg adjusted p-value threshold of 0.01 and a two-fold average change. Gene ontology (GO) enrichment analysis of upregulated genes upon PMA/ionomycin stimulation was performed using the *Metascape* web application [50].

#### Cell type annotation in 10X or HT Smart-seq3 data only

In addition to the integrative analysis, we performed cell type annotation separately on the 10X and HT Smart-seq3 data to evaluate whether T-cell subtypes could be captured by each platform independently. For comparison, the 10X data was down-sampled to match the cell count of the HT Smart-seq3 data for each sample. We then applied the same approaches used in the integrative analysis for cell clustering, marker gene identification, and cell type assignment. The only adjustment was the use of a higher resolution (1.5) in the *FindClusters()* function within Seurat for cell clustering.

#### Bulk RNA-seq data processing and analysis

A Nextflow pipeline was developed for quality control and gene expression quantification [51]. Quality control was performed using *FastQC* (v0.11.9) and *fastp* (v0.23.4) to ensure high-quality data [52]. Reads were mapped to the human reference genome GRCh38 using STAR (v2.7.6a) [53]. Gene expression count matrices were generated using *featureCounts* (Subread v2.0.1) [54]. Both genome reference sequence and gene annotation file were obtained from GENCODE (Release 38). The count data was normalized using the Trimmed Mean of M values (TMM) method implemented in the R package edgeR (v3.42.4). Differential gene expression analysis was conducted using the R package limma (v3.56.2).

## Electronic supplementary material

Below is the link to the electronic supplementary material.

Supplementary Material 1

## Data Availability

The datasets generated for this study are publicly available in the US National Center for Biotechnology Information Search (NCBI) Gene Expression Omnibus (GEO) repository under the accession numbers: GSE270917 (10X), GSE270928 (HT Smart-seq3), and GSE270511 (bulk RNAseq).
