# Supplementary material for: Single-cell sequencing of full-length transcripts and T-cell receptors with automated high-throughput Smart-seq3
Source: BMC Genomics. 2024 Nov 21;25:1127. doi: 10.1186/s12864-024-11036-0 (PMC11583680; doi:10.1186/s12864-024-11036-0)
Supplement: Supplementary file 1 — Supplementary Material 1 [file 12864_2024_11036_MOESM1_ESM.pdf]

Supplementary Information

Additional file 1:

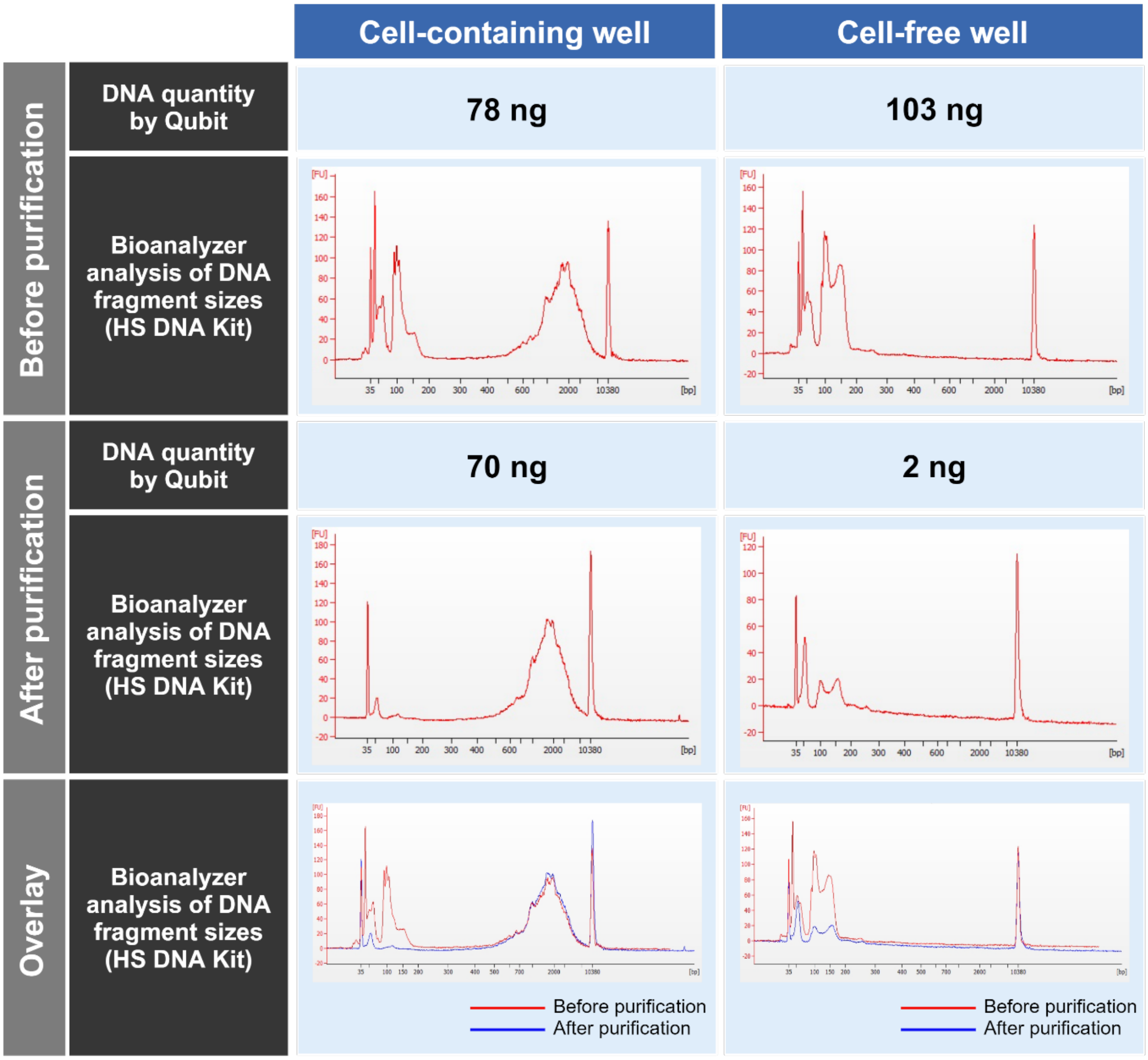

Figure S1 Comparison of cDNA quantification results using Qubit and Bioanalyzer for samples from cell-containing and cell-free wells, both before and after purification. This highlights the critical role of cDNA purification in achieving accurate and reliable quantification.

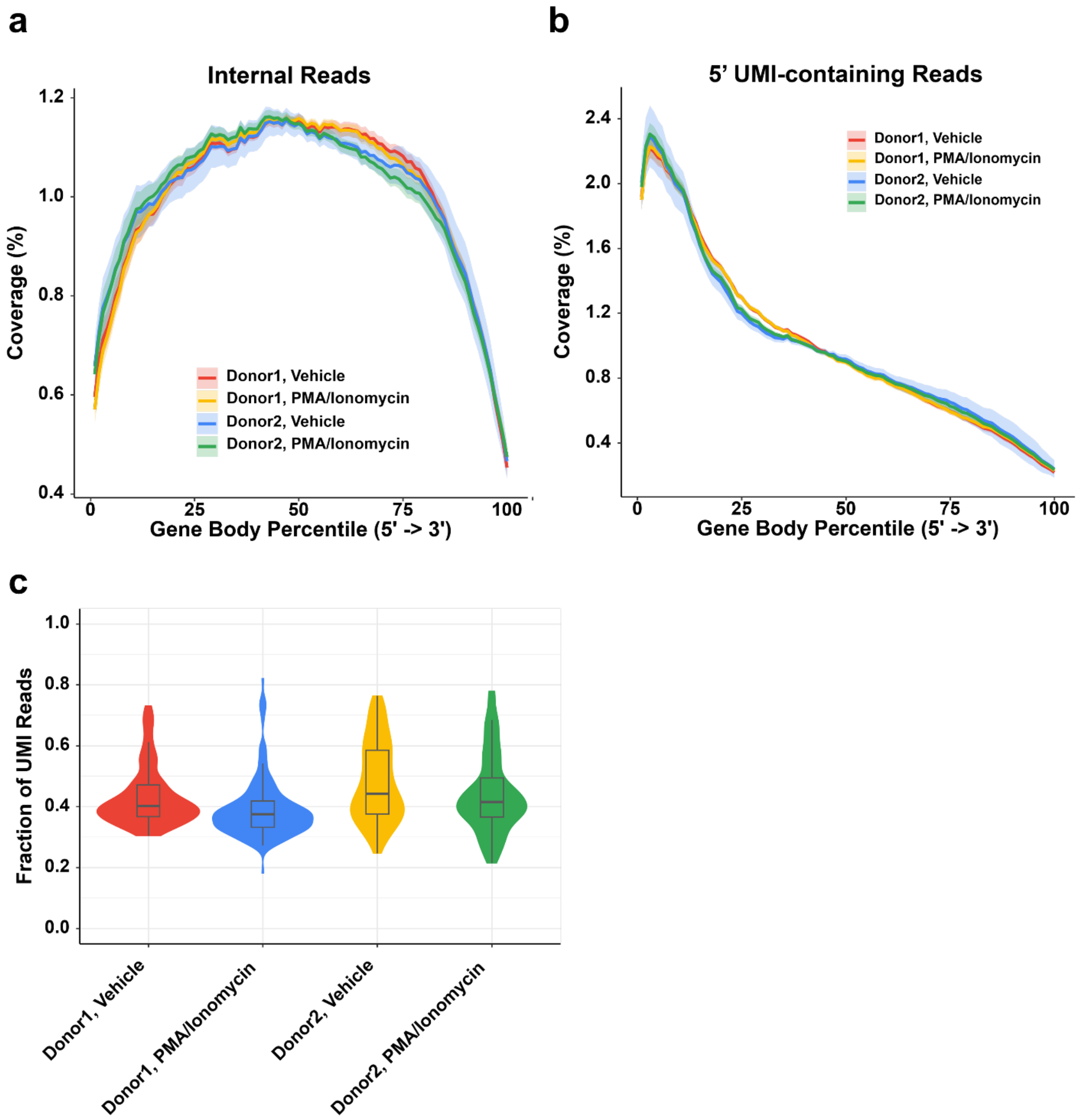

Figure S2 Statistics of internal reads and 5' UMI-containing reads in HT Smart-seq3 dataset. **a** Gene body coverage of internal reads. **b** Gene body coverage of 5' UMI-containing reads. **c** Fraction of 5' UMI-containing reads across four samples analyzed in this study.

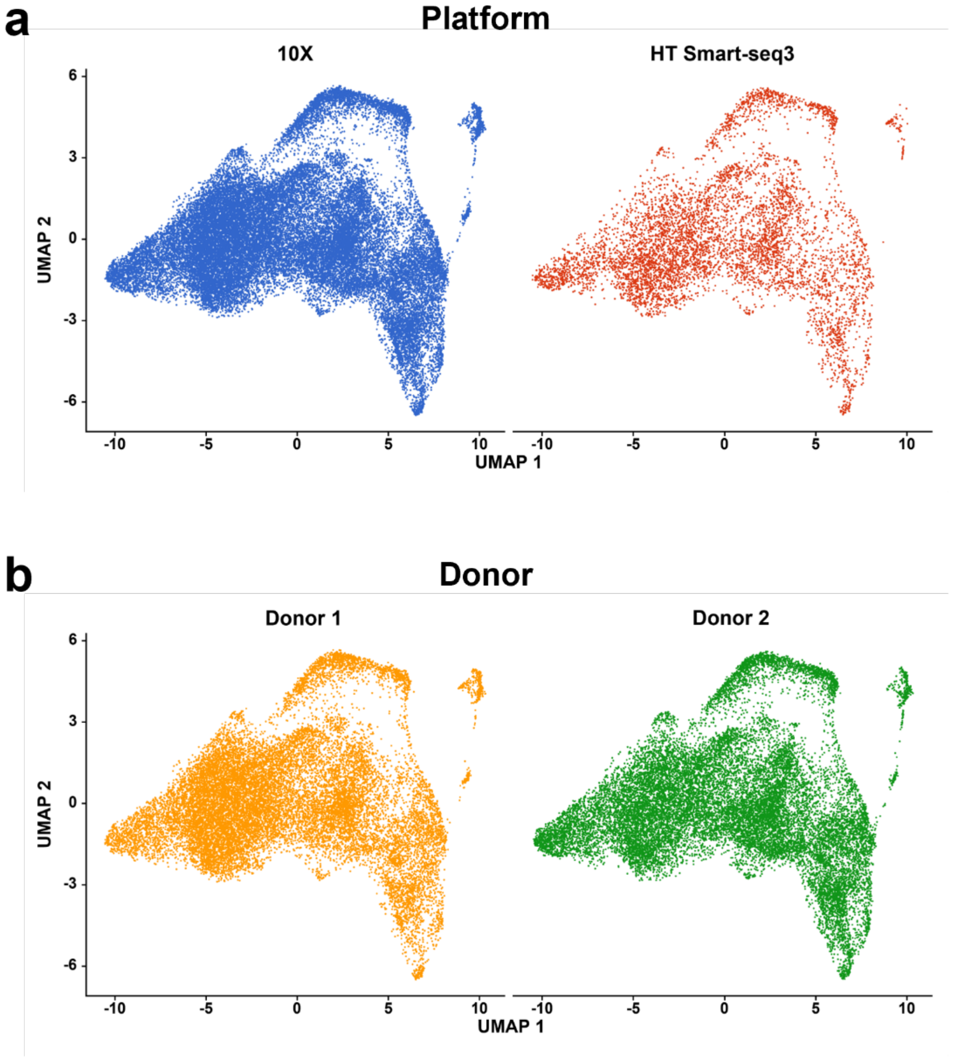

Figure S3 UMAP visualization of cells separated by **a** platform, and **b** donor, respectively.

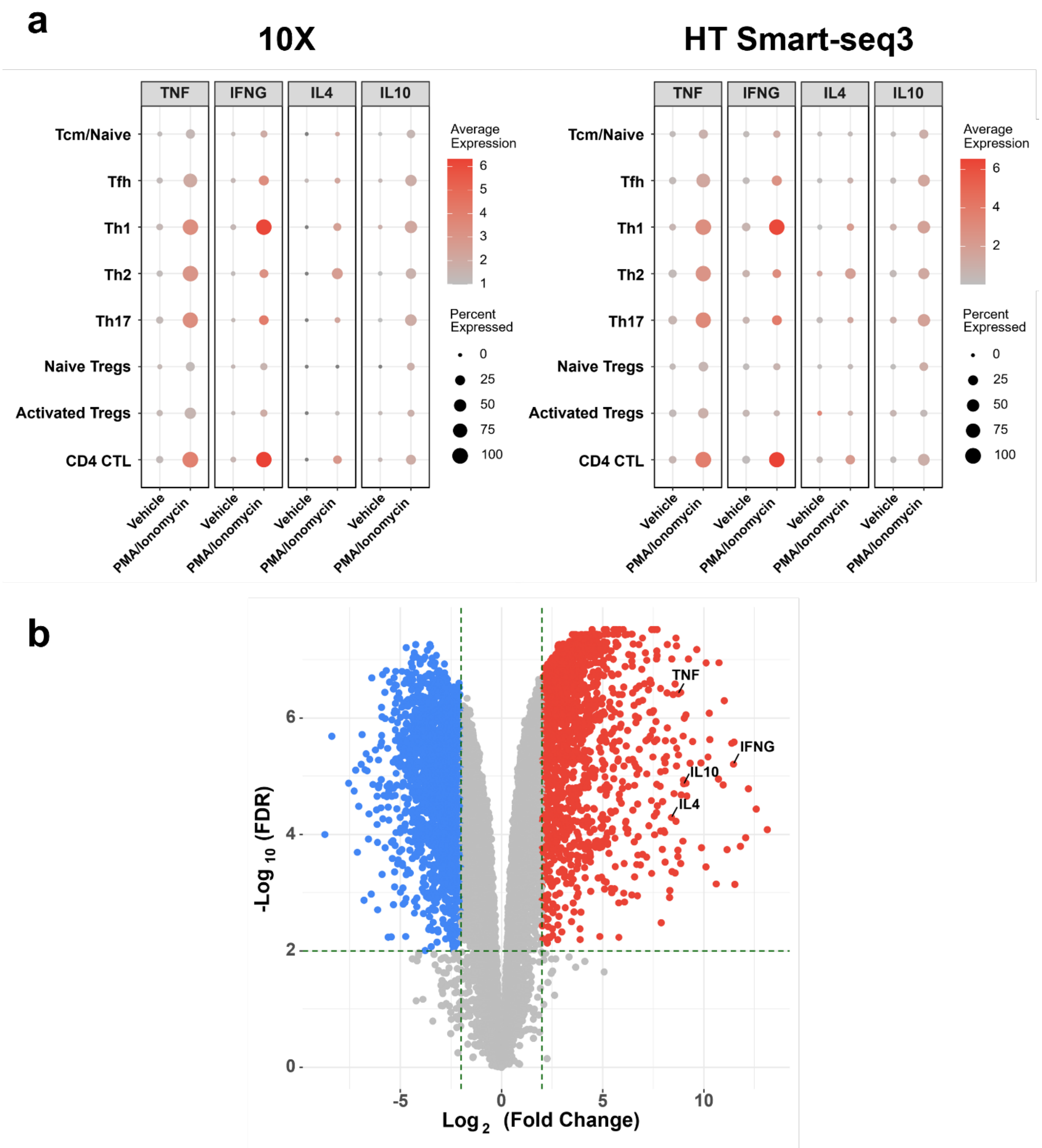

Figure S4 Expression of cytokines of interest in **a** 10X and HT-Smart-seq3, and **b** bulk RNA-seq, respectively. The upregulation of TNF, IFNG, IL4 and IL10 were observed in each T-cell subtype upon PMA/ionomycin stimulation.

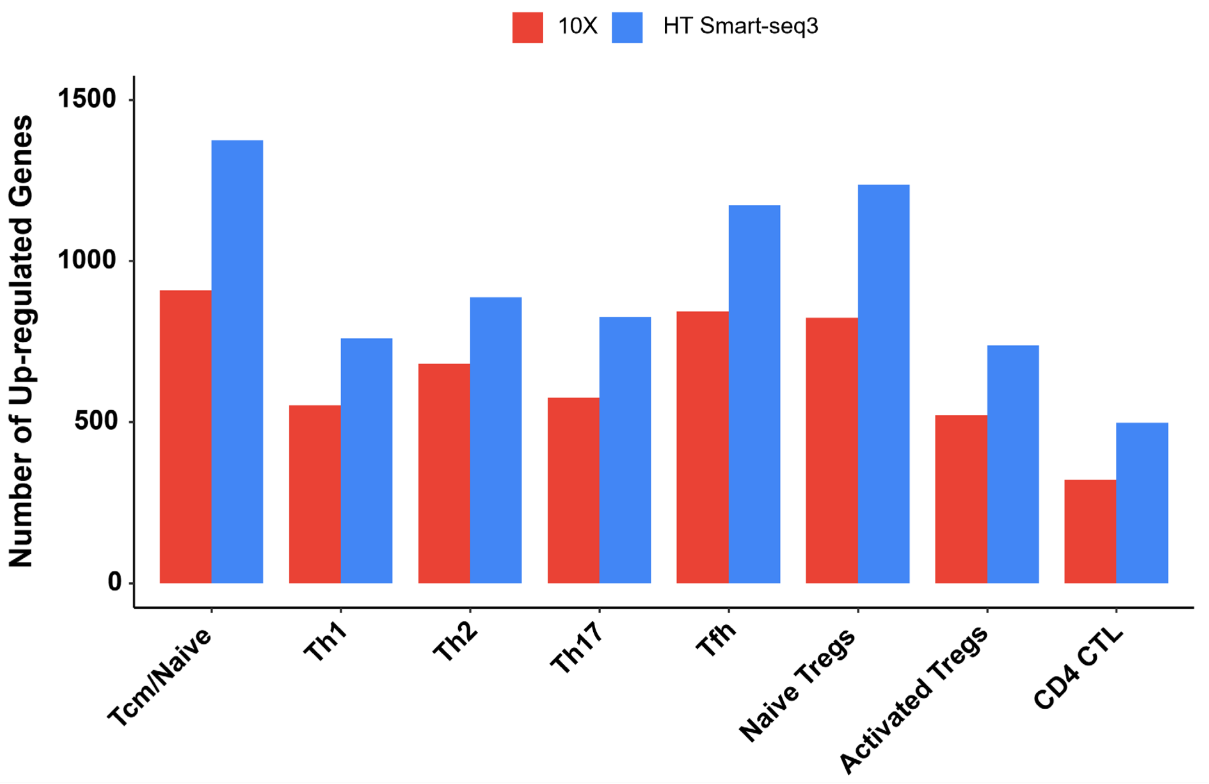

Figure S5 Down-sampling of 10X data to match HT Smart-seq3 cell count reveals that HT Smart-seq3 consistently detects more DEGs in each T-cell subtype.

a

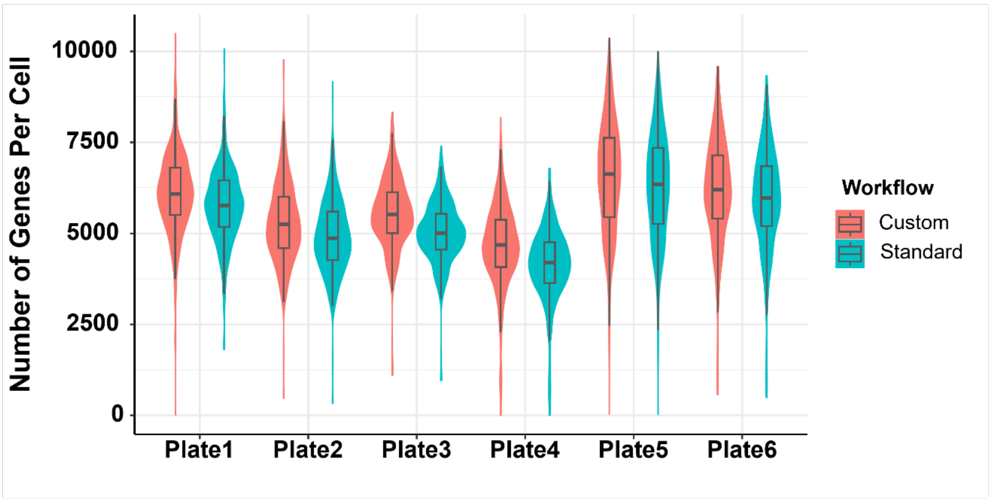

b

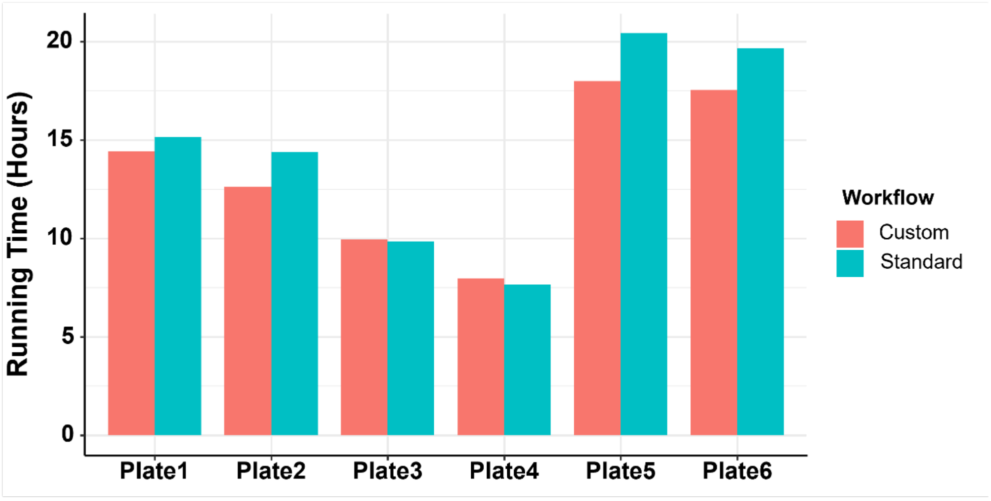

Figure S6 Comparison between our customized pipeline (Custom) and the best practice pipeline recommended by Hagemann-Jensen *et al.* (Standard). Six randomly selected plates were used for the evaluation. **a** Number of genes detected per cell for each plate. **b** Data processing time for cells from each plate. Both pipelines were run on Amazon EC2 r5n.4xlarge instance with 16 CPUs and 128 GiB of memory.

Additional file 2:

Table S1 Number of cells for each CD4+ T-cell subtype identified from the 10X and HT Smart-seq3 datasets.

	10X		HT Smart-seq3	
	Vehicle	PMA/ionomycin	Vehicle	PMA/ionomycin
Tcm/Naïve	9329	9386	1978	1739
Th1	615	1555	85	231
Th2	939	1169	133	188
Th17	752	1614	104	259
Tfh	1376	2558	324	519
Naïve Tregs	963	704	296	133
Activated Tregs	454	532	124	76
CD4 CTL	212	451	71	78
